# Current status of fish-borne zoonotic trematode infections in Gia Vien district, Ninh Binh province, Vietnam

**DOI:** 10.1186/s13071-015-0643-6

**Published:** 2015-01-13

**Authors:** Nguyen Manh Hung, Do Trung Dung, Nguyen Thi Lan Anh, Phan Thi Van, Bui Ngoc Thanh, Nguyen Van Ha, Hoang Van Hien, Le Xuan Canh

**Affiliations:** Institute of Ecology and Biological Resources (IEBR), Vietnam Academy of Science and Technology, 18 Hoang Quoc Viet St., Cau Giay district Hanoi, Vietnam; Department of Parasitology, National Institute of Malariology, Parasitology and Entomology (NIMPE), 245 Luong The Vinh St., Thanh Xuan district Hanoi, Vietnam; Department of Parasitology, National Institute of Veterinary Research (NIVR), 86 Truong Trinh St., Dong Da district Hanoi, Vietnam; Research Institute of Aquaculture No. 1 (RIA1), Dinh Bang, Tu Son, Bac Ninh, Vietnam

**Keywords:** Small trematode eggs, *Clonorchis sinensis*, Intestinal trematodes, Intermediate host, Aquaculture

## Abstract

**Background:**

Ninh Binh province is known as an endemic area of fish-borne zoonotic trematode (FZT) transmission in Vietnam. A cross-sectional study was conducted in Gia Minh and Gia Thinh communes of Gia Vien district, Ninh Binh province to investigate the infections with different stages of FZT in various host species.

**Methods:**

Faecal samples from 1,857 humans were examined for trematode eggs using the Kato-Katz method, while faecal samples from 104 dogs, 100 cats, and 100 pigs were examined using the Formalin-ethyl acetate concentration technique (FECT). A total of 483 specimens of freshwater fish, representing 9 species, were examined for metacercariae using the artificial digestion method. Three methods of cercarial detection (shedding, crushing and cutting) were applied for examination of 3,972 specimens of freshwater snails, representing 7 species. All relevant data e.g. location, sex, age group, animal species, and habitat were recorded for statistical analyses.

**Results:**

Helminth eggs were found in 65.5% of the human faecal samples, including 20.5% of faecal samples containing small trematode eggs. Infection with small trematodes differed among communes, age groups and sexes. Eggs of small trematodes were found in 32.7% of faecal samples from dogs, 49.0% from cats and 13.0% from pigs. The difference in prevalences and intensities were significant among species of animals but did not differ between the two communes. All fish species were infected with FZT, with an average prevalence of 56.1% and a mean intensity of 33.245 metacercariae per gram. Prevalence and intensity in fish differed significantly among cummunes and fish groups. Six species of zoonotic trematodes were identified. Metacercariae of the small liver fluke, *Clonorchis sinensis,* was only found in *Hemiculter leucisculus*. A total of 9 specimens from two snail species, *Melanoides tuberculata* and *Bithynia fuchsiana,* were infected with trematodes and four cercarial types were detected in the study sites.

**Conclusions:**

We conclude that Gia Minh and Gia Thinh communes are continuing to be hot-spot endemic areas of FZT and other helminths infections where the habit of eating raw fish by the local people is still present.

## Background

Fish-borne zoonotic trematodes (FZT) are transmitted by fish and fish products, and pose a major public health problem [1-3]. People become infected with FZT after ingesting raw or undercooked freshwater fish containing infective metacercariae [3-5]. Recently, a total of 59 FZT species, which are known to parasitize humans, are listed [6]. All species can be divided into two groups, the first being the small liver flukes (Opisthorchiidae: 12 species), and the second the minute intestinal flukes (Heterophyidae: 36 species, Echinostomatidae: 10 species and Nanophyetidae: 1 species) [6]. Adults of the small liver flukes parasitize the liver (bile ducts and gall bladder) of their definitive host, where they can cause serious diseases in humans. Cholangitis, choledocholithiasis, pancreatitis, and cholangiocarcinoma are the major clinical problems associated with chronic infections [7,8]. Individuals with light infections usually show no symptoms. Intestinal flukes are generally not of considerable clinical importance compared to the liver flukes, but several species may cause significant pathology sometimes fatal; in the heart, brain, and spinal cord of humans [9-13]. Eggs of intestinal flukes are difficult to differentiate from those of liver flukes, often causing misdiagnosis and inaccurate estimates of the prevalence of both trematode groups [14,15].

The worldwide number of people currently infected with small liver flukes only exceeds 45 million [4]. Among them, 35 million people are infected with *Clonorchis sinensis*, including 15 million Chinese [16,17], 10 million people are infected with *Opisthorchis viverrini* [4] and 1.6 million people are infected with *O. felineus* [18] including 1.5 million people in the former Union of Soviet Socialist Republics [19]. The estimates of at-risk populations for clonorchiasis and opisthorchiasis are 601 million and 80 million, respectively [4]. In cases of small intestinal flukes, an estimated 40 to 50 million people are infected with one or several species [4,20], with more than half a billion at risk of infection [21,22]. In Vietnam, 12 FZT species have been found in humans and mammals, including 3 small liver fluke species and 9 minute intestinal fluke species [6]. Seven FZT species have only been detected in humans: *Clonorchis sinensis*, *Opisthorchis viverrini* (Opisthorchiidae), *Centrocestus formosanus*, *Haplorchis pumilio*, *H. taichui*, *H. yokogawai*, *Stellantchasmus falcatus* (Heterophyidae). Estimates of people infected with small liver flukes were 2 million [4], with unknown millions infected with intestinal flukes. The number of infected people with intestinal flukes is believed to be higher than the number of people infected with small liver flukes [23].

We conducted a study in Gia Vien district, Ninh Binh province from August 2011 to February 2012. This area has a proverb “Nắng gỏi, mưa cày” meaning the local people delight in eating raw fish during the dry season and dog meat in the rainy season. The habit of eating raw fish by people here is the only reason to explain the high prevalence of small liver fluke infections, according to previous surveys [24-26]. Our survey aimed to clarify the parasitological status of FZT infections in different hosts, including first intermediate hosts (snails), second intermediate hosts (fishes), and the definitive hosts (humans and mammals).

## Methods

### Study site

Two communes, Gia Minh and Gia Thinh in Gia Vien district, Ninh Binh province, were selected for cross-sectional surveys. These communes are known as heavily clonorchiasis-endemic areas in Northern Vietnam. The population in Gia Thinh commune was 7,615, including 1,228 families, while in Gia Minh commune the population was 2,791 with 512 families. Because of flooding in the rainy season caused by Hoang Long River, farmers in both communes do not have fish ponds integrated into their farming systems, (e.g. pig farming, vegetable garden) as other rural areas. Most of the residents are living on rice agriculture; however, some farmers rely on fish cage cultures in the rivers, dams, and lakes.

### Examination of human and domestic animal fecal samples

Households in these two communes were randomly selected from a list provided by community authorities. In each selected household, all members of the family, age 2 years or older, were chosen for the study. The trained personnel gave labeled plastic bags to the selected persons older or equal to 9 years of age and instructed them on how to collect and store their fecal sample until it was returned the next day. For instances where participants were under the age of 9, the proxy helped in the collection of the fecal sample. The leader of the household also collected fecal sample from domestic animals (dogs, cats and pigs) and returned them together with the human fecal samples. Human fecal samples were labeled with the participant’s name, age, address, and date of stool collection. Animal fecal samples were labeled with the name of the householder, the animal species, ordinal number if the household had more than one individual of a species, address, and date of stool collection. Human and animal fecal samples were examined by the National Institute of Malariology, Parasitology and Entomology (NIMPE) and National Institute of Veterinary Research (NIVR), separately. Fecal samples were kept at 4°C in the laboratory. A total of 1,857 people, 104 dog, 100 cats and 100 pigs were examined in both communes.

Kato-Katz thick smear method was applied to examine human fecal samples. Each fecal sample was prepared for one Kato-Katz smear and analyzed using a standard kit provided to NIMPE by the World Health Organization and originally obtained from Vestegaard Frandsen Pvt. Ltd. (New Delhi, India). Fecal slides were examined by light microscopy (×400) to identify helminth eggs. Identification of helminthic eggs based on morphological features detailed by WHO [27]. Fecal samples of animals were examined using a Formalin-ethyl acetate concentration technique (FECT) [28] for finding only trematode eggs. All trematode eggs which had a size smaller than 50 μm were designated “small trematode eggs” [23,29,30]. The infection intensity of trematode eggs was calculated for each sample.

### Fish sampling and examinations

Fish samples were collected from different water bodies, e.g. rivers, lakes, dams, and small canals, and were divided into two groups. The first group represented cultured fishes and the second wild-caught fishes. The term “wild-caught fish” refers to fish collected outside the aquaculture systems [31]. The culture fishes were collected from cage cultures, at the study sites, at one site in the Gia Thinh commune and 5 sites in the Gia Minh commune. The wild-caught fishes were collected from 15 different locations in each commune. Fishes in cages, dams and lakes were collected using a cast net, used in each of the four corners of each water body [31]. All fish caught from the four throws were used for examination. For fish living in rivers and canals, dip-nets were used for collections. In each location, the dip-net was used 5 times. The period of rest between dip-net use was 30 minutes. A total of 483 fish, belonging to 9 species, were collected and examined, including 172 culture fish specimens. After collecting, fish samples were preserved on ice and transported, same day, to the Center for Environment and Disease Monitoring in Aquaculture (CEDMA), RIA1. In the laboratory, fish were maintained at 4°C for a maximum of 5 days until processed. Each fish was examined using an artificial digestion method, described by WHO [32] and modified by Tran et al. [33]. Small fishes (less than 200 g in weight) were ground and digested whole, while only 50 g sub-sample from larger fishes (more than 200 g) were ground, mixed, and digested for recovery of metacercariae. Metacercariae of each type were counted and recorded.

Metacercariae were identified according to morphological features detailed by Pearson & Ow-Yang [34], Scholtz et al. [35] and Kaewkes [36]. Infection intensities of metacercariae were calculated for each sample.

### Snail sampling and examination

Thirty locations, representing all available habitats in each commune, were selected for snail collecting. Drainage canals nearby households were intensely sampled, with 15 sites, due to the heavy risk factor for food-borne zoonotic trematode infections associated with them [37]. Other habitats included rivers (2 sites), lakes (2 sites), dams (2 sites) and rice fields (9 sites). Rice fields were selected as far away as possible from one another to insure that all hamlets were represented at least once.

Snails were collected during the morning hours for 20 minutes per site by scooping [37,38] and/or hand-picking, transferred to plastic containers and transported alive to the laboratory where they were identified according to keys by Brandt [39] and Dang [40].

All snails, depending on their size, were examined for trematode infection using one or more of the following methods: shedding, crushing, and/or cutting. For the shedding method smaller snails were placed individually in small plastic containers with 5 ml of water each and left for 24 hours to shed cercariae. Snails were checked for shedding in the late afternoon and again the following morning. This procedure can be used for larger snails except they should be kept in larger containers with more water. The advantage of this method is the ability to obtain clean cercariae. The crushing method involves crushing whole snails between two glass plates while the cutting method (only done on large snails with rigid shells) involved cutting open the shell and transferring tissue parts and hemolymph to a glass slide. When checked under a stereomicroscope, several stages of trematode larvae (cercaria (old and young), sporocysts, and rediae) could be observed. Cercarial groups were identified to major types according to the keys of Ghihencinskaja [41], Shell [42] and other available references; more specific identification was not made.

### Statistical analysis

Data were entered into an Excel database (Microsoft Corporation, Redmond, WA, USA) and analyzed using statistical analysis software (STATA 12; StataCorp LP, College Station, TX, USA).

Trematode egg count data from human samples were transformed to binomial data. Cases with infection of any FZT species were coded to “1” and uninfected cases were coded to “0”. Occurrence (present/absence) of FZT in humans was analyzed using logistic regression, with commune, sex, and age group as predictors after adjusting for possible clustering within families and hamlet [43]. The age of participants was divided into three categories: under working-age group (<19), working-age group (19–59), and over working-age group (>59). Similarly, infections in domestic animals and fish were analyzed using logistic regression, with species or fish group and commune as predictors, while infections in snails used snail species, habitat, and commune as predictors.

Intensity of FZT infections in humans, domestic animals, and fish was analyzed in a similar fashion, using negative binomial regression and reported as count ratios, i.e. in generalized linear model with log-link function [44]. The ancillary parameter was estimated using full maximum likelihood estimation.

Differences with p-values below 0.05 were considered significant.

### Ethical aspects

The human study was approved by NIMPE, while NIVR approved the study of domestic animals. Before the start of the study, all selected households had been given information about the study and volunteered to participate in the study. All infected cases (both human and animal) were treated with anthelmintic free of charge at the conclusion of the study.

## Results

### Helminth infection in humans

Results for helminth and small trematode infections in humans using fecal egg examinations are summarized in Table 1 and Table 2. A total of 1,217 persons were infected with helminths (65.5%). Eggs of small trematodes were found in 20.5% of fecal samples. Nematode eggs of whipworm (*Trichuris trichiura*), roundworm (*Ascaris lumbricoides*), hookworm and other parasites were detected in 45%, 29.2%, 1.7% and 0.9% of fecal samples, respectively. Multiparasitism was common in the study sites, with 36.1% of participants having eggs of more than one species or type of eggs.

**Table 1:** **Helminth infections in humans living in Gia Minh and Gia Thinh communes**

**Helminths**	**Gia Minh (no. exam: 895)**	**Gia Thinh (no. exam: 962)**	**Total examination: 1,857**
	**No. positive (prevalence in %)**	**No. positive (prevalence in %)**	**No. positive (prevalence in %)**
Small trematodes	84 (9.4)	297 (30.9)	381 (20.5)
*Ascaris lumbricoides*	410 (45.8)	132 (13.7)	542 (29.2)
*Trichuris trichuira*	547 (61.1)	389 (40.4)	836 (45.0)
Hookworm	25 (2.8)	7 (0.7)	32 (1.7)
Other	5 (0.6)	12 (1.2)	17 (0.9)
Total infection	685 (76.5)	532 (55.3)	1,217 (65.5)

**Table 2:** **Prevalence of small trematode infections in relation to age group and sexes**

**Commune**	**Age group**	**Male**				**Female**			
		**No. examined**	**No. positive**	**Prevalence (%)**	**Mean intensity (range)**	**No. examined**	**No. positive**	**Prevalence (%)**	**Mean intensity (range)**
Gia Minh	<19	142	2	1.4	48 (48–48)	163	6	3.7	204 (24–696)
	19 - 59	162	37	22.8	245.8 (24–2,760)	271	20	7.4	328.8(24–1,920)
	>59	61	15	24.6	297.6 (24–1,560)	96	4	4.2	156 (24–480)
Gia Thinh	<19	82	11	13.4	24.3 (1–252)	68	7	10.3	5.9 (1–30)
	19 - 59	275	148	53.8	33.5 (1–810)	391	75	19.2	31.3 (1–1,200)
	>59	54	28	51.9	55.7 (1–470)	92	28	30.4	107.1 (1–1,520)
Total		776	241	31.1		1081	140	13.0	

The prevalence and intensity of small trematodes in Gia Thinh commune were 4.62 times (p < 0.001) and 1.2 times (p = 0.009) higher than those in Gia Minh commune, respectively. Infection with small trematodes also differed between age groups and sex. The rate of infection and intensity in men were 2.99 and 1.82 times greater than in women (p < 0.001). The working-age group and over working-age group had prevalences 6.79 and 6.81 times higher than in the under working-age group (p < 0.001). Similarly, the intensity of small trematode infections in the working-age group and over working-age group were 1.8 and 2 times higher than in the under working-age group (p < 0.001). There was no significant difference in small trematode infections between working-age group and over working-age group.

Infections with hookworms did not differ significantly by sex, age group, or commune (p > 0.05). The prevalence and intensity of *Trichuris trichiura* in Gia Minh commune were 2.5 and 9.4 times higher than those in Gia Thinh commune (p < 0.001), however, there were no significant differences among age groups and sexes. In cases of *Ascaris lumbricoides*, infections differed significantly among commune, sex, and age group. Prevalence and intensity of round worm infections in Gia Minh commune were 3.8 and 11.2 times higher than those in Gia Thinh commune (p < 0.001). The under working age group had infection prevalences 1.7 and 1.9 times higher than in the working-age and over working-age groups (p < 0.001), however there were no significant differences between the working-age and over working-age groups. Similarly, the intensity of round worm infections in the under working-age group was higher than in the working-age and over working-age groups (p < 0.001). The odds ratios were 2.9 and 3.0, respectively. Women had prevalence and intensity of round worm infection 1.6 (p = 0.001) and 1.4 (p = 0.004) times higher than men.

### Prevalence and intensity of small flukes in animals

Results of examination of animal samples for small trematodes are summarized in Table 3. Prevalence of small fluke infections was 32.7% in dogs, 49.0% in cats, and 13.0% in pigs. The difference in prevalence and intensity between dogs, cats and pigs was significant (p < 0.05). Prevalence of infection was highest in cats and lowest in pigs, while the intensity was highest in dogs and lowest in pigs. There were no significant differences in prevalence and intensity of small trematode infections between the two communes for dogs, cats, or pigs (p > 0.05).

**Table 3:** **Prevalence and intensity of FZT infections in domestic animals in Gia Minh and Gia Thinh communes**

**Commune**	**Dogs**				**Cats**				**Pigs**			
	**No. of samples**	**No. of positive samples**	**Prevalence (%)**	**Mean intensity (range)**	**No. of samples**	**No. of positive samples**	**Prevalence (%)**	**Mean intensity (range)**	**No. of samples**	**No. of positive samples**	**Prevalence (%)**	**Mean intensity (range)**
Gia Minh	51	18	35.3	258.8 (51–628)	40	23	57.5	125.4 (58–487)	57	4	7.0	16.3 (8–25)
Gia Thinh	53	16	30.2	229.6 (84–592)	60	26	43.3	132.2 (73–520)	43	2	4.7	13.5 (10–17)
Total	104	34	32.7		100	49	49.0		100	6	6.0	

### Infection status of FZT in fish

A total of 483 fish, representing nine species, were examined for FZT. Among them, the Crucian carp, tilapia, and common carp were the dominant species, comprising 68.9% of the total number of examined fishes (Table 4). All fish species were infected by FZT at different prevalences. The average prevalence was 56.1% with a mean intensity of 33.245 metacercariae per gram (Table 5). Several fish species showed high prevalence of infections, i.e. *Anabas testudineus* (96.8%), *Channa striata* (85.7%) and *Carassius auratus* (79.6%). The lowest prevalence was found in *Labeo rohita* (11.4%).

**Table 4:** **Prevalence of FZT in various fish species**

**Fish species**	**Culture fish**			**Wild-caught fish**			**Total**		
	**No. of samples**	**No. of positive**	**Prevalence (%)**	**No. of samples**	**No. of positive**	**Prevalence (%)**	**No. of samples**	**No. of positive**	**Prevalence (%)**
Silver carp (*Hypophtalmychthys molitrix*)	32	14	43.8				32	14	43.8
Common carp (*Cyprinus carpio*)	42	8	19.0	14	7	50.0	56	15	26.8
Rohu (*Labeo rohita*)	35	4	11.4				35	4	11.4
Tilapia (*Oreochromis niloticus*)	63	19	52.8	28	4	14.3	91	23	25.3
Sharpbelly (*Hemiculter leucisculus*)				8	4	50.0	8	4	50.0
Snakehead murrel (*Channa striata*)				7	6	85.7	7	6	85.7
Crucian carp (*Carassius auratus)*				186	148	79.6	186	148	79.6
Ray-finned fishes (*Rasborinus hautus*)				37	18	48.6	37	18	48.6
Climbing perch (*Anabas testudineus*)				31	30	96.8	31	30	96.8
Total	172	45	26.2	311	217	69.8	483	271	56.1

**Table 5:** **Prevalence and intensity of FZT in fish stratified by fish groups and communes**

**Commune**	**Fish group**	**No. examined**	**No. positive**	**Prevalence (%)**	**Mean intensity (range)**
Gia Minh	Wild-caught	178	106	59.6	33.823 (0.004-499.95)
	Culture	156	42	26.9	0.699 (0.004-57.804)
Gia Thinh	Wild-caught	133	84	63.1	63.741(0.009-826.364)
	Culture	16	3	1.8	0.007 (0.009-0.01)
Total		483	271	56.1	33.245(0.004-826.364)

The prevalence and intensity of FZT in fish from Gia Thinh commune were 2.5 and 1.5 times higher than in fish from Gia Minh commune (p < 0.005), respectively. The wild-caught fish group had prevalence of FZT 5.1 times higher than in the culture fish group (p < 0.001). Similarly, the intensity of FZT metacercariae in the wild-caught fish group was 1.8 times greater than in the culture fish group.

A total of six species of zoonotic trematodes was identified, including *Haplorchis pumilio*, *H. taichui*, *H. yokogawai*, *Centrocestus formosanus*, *Procerovum varium* (Heterophyidae), and *Clonorchis sinensis* (Opisthorchiidae) (Table 6). Numerous metacercariae could not be identified to genus or species because they died or were too young; therefore, they were listed as unidentified. Metacercariae of the liver fluke, *C. sinensis*, was only found in the sharpbelly, with a prevalence of 12.5%. Other intestinal flukes, e.g. *Haplorchis* spp. and *P. varium*, were commonly found in most examined fish species, while *C. formosanus* was recovered from the Crucian carp and ray-finned fishes. Multiparasitism was common in the examined fish. Five fish species were infected with more than one FZT species.

**Table 6:** **Occurrence of FZT metacercariae in different fish species**

**Fish species**	**prevalence (no. positive/no. sample)**	**FZT species found**				
		***Clonorchis sinensis***	***Haplorchis*** **spp.**	***Procerovum varium***	***Centrocestus formosanus***	**Unidentified**
Silver carp (*Hypophtalmychthys molitrix*)	48.8(14/32)	0	12	0	0	4
Common carp (*Cyprinus carpio*)	26.8 (15/56)	0	13	0	0	0
Rohu (*Labeo rohita*)	11.4 (4/35)	0	4	1	0	0
Tilapia (*Oreochromis niloticus*)	25.3 (23/91)	0	21	2	0	6
Sharpbelly (*Hemiculter leucisculus*)	50.0 (4/8)	1	3	0	0	0
Snakehead murrel (*Channa striata*)	85.7 (6/7)	0	6	1	0	0
Crucian carp (*Carassius auratus)*	79.6 (148/186)	0	133	12	3	49
Ray-finned fishes (*Rasborinus hautus*)	48.6 (18/37)	0	15	3	1	4
Climbing perch (*Anabas testudineus*)	96.8 (30/31)	0	27	26	0	0
Total	56.1 (271/483)	1	234	45	4	63

### Diversity of snails and trematode infections

A total of 3,972 snails, representing 7 species, were collected in both Gia Minh and Gia Thinh communes (Table 7). *Bithynia fuchsiana*, *Melanoides tuberculata*, *Angulyagra polyzonata* and *Lymnaea viridis* were commonly found. They comprised 91.2% of the total examined snail specimens. *Bithynia fuchsiana* was particularly common in rice fields and small canals, while *Lymnaea* spp. were commonly found in rice fields and on floating aquatic macrophytes in lakes. *Melanoides tuberculata*, *A. polyzonata*, and *P. canaliculata* were present in all habitats, while *S. aeruginosa* were found in rivers, lakes, and dams.

**Table 7:** **Prevalence of infected snails in the study sites**

**Snail species**	**Gia Minh commune**		**Gia Thinh commune**	
	**No. snails collected**	**No. snails infected**	**No. snails collected**	**No. snails infected**
*Melanoides tuberculata*	432	6	326	1
*Bithynia fuchsiana*	794	1	1100	1
*Angulyagra polyzonata*	207	0	179	0
*Sinotaia aeruginosa*	89	0	103	0
*Lymnaea viridis*	324	0	261	0
*L. swinhoei*	51	0	77	0
*Pomacea canaliculata*	12	0	17	0
Total	1909	7	2063	2

Among the 7 snail species, only *M. tuberculata* and *B. fuchsiana* were emitting cercariae, with prevalences of 0.92% and 0.11%, respectively. All infected snails were found in small canals. There was no significant difference in prevalence of trematodes in snails between the two communes (p = 0.074). Four cercarial types were recorded. *Bithynia fuchsiana* only released Echinostome cercariae, while *M. tuberculata* released all 4 cercariae types (Table 8).

**Table 8:** **Cercariae groups found infecting the first intermediate host**

**Cercariae**	**Snail species**	
	***M. tuberculata***	***B. fuchsiana***
Xiphidiocercariae	2	0
Echinostome cercariae	1	2
Parapleurolophocercariae	2	0
Furcocercariae	2	0

## Discussion

Small trematode eggs, all <50 μm long, were presumed to be either those of *C. sinensis* or of intestinal trematodes of the family Heterophyidae or Echinostomatidae in Vietnam. However, differentiation was not considered reliable by light microscopy. Freshwater fish are the major source of infections with intestinal and liver trematodes [45]. Other flukes are transmitted through plants, snails, crustaceans, amphibians, reptiles and insects [45]. So, all small trematode eggs found in infected humans in study sites, are presumably FZT.

Prevalence of fluke infections in animals in Gia Minh and Gia Thinh communes did not differ, while the chance of people in Gia Thinh becoming infected was 4.62 times higher than for individuals in Gia Minh. Conversely, the prevalence of nematode infections in people in Gia Minh was higher than in Gia Thinh. This difference could be explained by the higher frequency of eating raw fish and other raw foods by the local people. Unfortunately, during this study we did not conduct a survey on the eating habits of the local people.

Infection rates of small trematodes were higher for persons >18 years of age and no difference was observed between the working-age group and the over working-age group, a pattern believed to be caused by longer exposure and accumulation of flukes because some trematode species have a long life span [1,46]. Mas-Coma & Bargues [8] mentioned that the longevity of the parasite depends on the host-parasite compatibility and the tolerance of the host. In case of small liver flukes, e.g. *C. sinensis*, adult stage can survive 15–25 years in humans, but an extreme longevity of over 26–40 years has also been deduced [8]. However, the longevity of small intestinal flukes in humans is not well documented, therefore accumulation of flukes as an explanation for age-related infection patterns is speculative [23]. The infection rates of men with trematodes was higher than of women, and is likely associated with male-oriented social gatherings, during which they consume raw fish or fish products [23,47]. The prevalence of nematode infections in the under working-age group were higher than in the two other age groups. Additionally, women had a higher incidence of nematode infections than men. This could be a result of the women and children coming into contact with contaminated soil, either through playing or working, more often than men, leading to greater chance of soil transmitted helminth larvae.

Small trematode infections were common in cats, dogs with lower levels in pigs, in both Gia Minh and Gia Thinh communes. Cats and dogs are intentionally fed raw fish by farmers, and are capable of scavenging for raw fish from local ponds and surrounding canals. Pigs, however, become infected only if their owners feed them raw fish or fish waste [48].

At both study sites, local people use domestic animal manure (in some cases, human manure) as fertilizer for the rice fields. This is a significant risk factor for FZT infection, because the eggs in the manure are viable in the wet environment of the rice fields, where snails and fishes are available as the first and the second intermediate hosts. In addition, flooding of the Hoang Long River during the rainy season inundates the rice fields, allowing the trematode eggs present in the animal/human manure to enter the local water system, likely increasing the prevalence and intensity of infected snails.

The diversity and prevalence of FZT infections were found to be high in fishes, although metacercariae of *Clonorchis sinensis* was only found in the sharpbelly (*Hemiculter leucisculus*). Local people in the two study sites normally use several freshwater fish species: i.e. silver carp (*Hypophthalmichthys molitrix*), grass carp (*Ctenopharyngodon idellus*), rohu (*Labeo rohita*), and mrigal (*Cirrhinus mrigala*) to prepare raw fish dishes. Fish are filleted, sliced, and soaked twice in saline solution (1-3%) before being mixed with rice powder and consumed with herbs and sauce [49,50]. Tran et al. [50] found that 11.8% dishes in restaurants from Nam Dinh province (nearby Ninh Binh province where our study was conducted) were infected with FZT, and freshwater fish were more often infected (39.4%) than brackish water fish (16.0%). The odds of FZT infection was 2.3 times higher for humans eating raw fish than for those who did not eat raw fish [49]. Metacercariae still remain viable in the prepared fish, even with harsh conditions, i.e. Wykoff [51] found that metacercariae die after 7 hours of desiccation at room temperature, 3 minutes at 65°C or 2.5 hours at 39–40°C when removed from its host fish. Metacercariae within fish meat under refrigeration remain viable after 40 days, and apparently 50% were still viable after 50 days when kept at 3–6°C. Fan [52] studied the effect of freezing or salting of fish on viability of metacercariae of *C. sinensis*. He found that metacercariae from freshwater fish stored at −12°C for 10–18 days or −20°C for 3–7 days or 5–7 days at heavy salt concentration (3 g salt/10 g fish) remained viable and infective. However, it appears that refrigeration or keeping fish in salt for longer periods (2 months) may be suitable for prevention of infection. Local people within the study understood the risk of becoming infected with liver flukes when eating raw fish from information provided by the government through mass media under “national helminthiasis control activities” program [40]. However, they still maintain the habit of consuming raw fish.

The prevalence and intensity of FZT infections in the culture fish group were lower than in the wild-caught fish group. It could be explained by the different density of snails in various environments. Snail density is considerably lower in cage culture than in small canals, lakes, dams, and rivers. In the cage cultures, fishes consume snails, even if they do not feed on snails, they could cause damage or disturbance when probing various items (including snails) for suitability as food [36]. The presence of the black carp, a snail predator [6,53-55], in cages seems to reduce snail density, although the actual presence of the black carp in these cages was not confirmed, just based on interviewing farmers.

Cercariae of the Opisthorchiidae belong to the pleurolophocercarial type. These cercarial type were not found in our survey while Kino et al. [24] found 13.3% of *Melanoides tuberculata* infected with *Clonorchis sinensis* in the similar study sites. Nguyen [26] also found that the prevalences of infection with *C. sinensis* in *Parafossarulus striatulus* and *M. tuberculata* were 5.1% and 10.2%, respectively. The identification of cercariae of *C. sinensis*, however, based on morphological characteristics alone may not be accurate, i.e. species of three trematode families, the Heterophyidae, Opistorchiidae and Cryptogonimiidae, possess pleurolophocercous cercaral type [42]. For this reason, the results of the two references above have been questioned by others [36]. Hence there is need to include molecular tools for identification of both snails and their trematode infections [56].

## Conclusions

In general, Gia Minh and Gia Thinh communes are still endemic areas of FZT and other helminth diseases, although local people are aware of the risks of eating raw fish. Any strategy for controlling helminth diseases must make local people realize the harmfulness of parasites, especially FZT, and they should break the habit of eating raw fish.
